# Supplementary material for: Multifactorial analysis of terminator performance on heterologous gene expression in Physcomitrella
Source: Plant Cell Rep. 2024 Jan 22;43(2):43. doi: 10.1007/s00299-023-03088-5 (PMC10800305; doi:10.1007/s00299-023-03088-5)
Supplement: Supplementary file 1 — Supplementary file1 (DOCX 1085 KB) [file 299_2023_3088_MOESM1_ESM.docx]

­­­­­­­Electronic Supplementary material

**Multifactorial analysis** **of terminator performance on heterologous gene expression in Physcomitrella**

Paul Alexander Niederau^1^, Pauline Eglé^1^, Sandro Willig^1^, Juliana Parsons^1^, Sebastian N.W. Hoernstein^1^, Eva L. Decker^1^, Ralf Reski^1, 2,*^

^1^ Plant Biotechnology, Faculty of Biology, University of Freiburg, Freiburg, Germany

^2^ Signalling Research Centre BIOSS and CIBSS, University of Freiburg, Freiburg, Germany

*Corresponding author: ralf.reski@biologie.uni-freiburg.de

ORCID:

P.A.N. 0009-0004-0194-8570

P.E. 0009-0002-1115-5828

S.W. 0009-0001-2816-4918

J.P. 0000-0001-6261-2342

S.N.W.H. 0000-0002-2095-689X

E.L.D. 0000-0002-9151-1361

R.R. 0000-0002-5496-6711

Supplementary tables S4, S5, and S6 are deposited on Zenodo (doi.org/10.5281/zenodo.8083448).

**Supplementary file S1 - Vector maps**


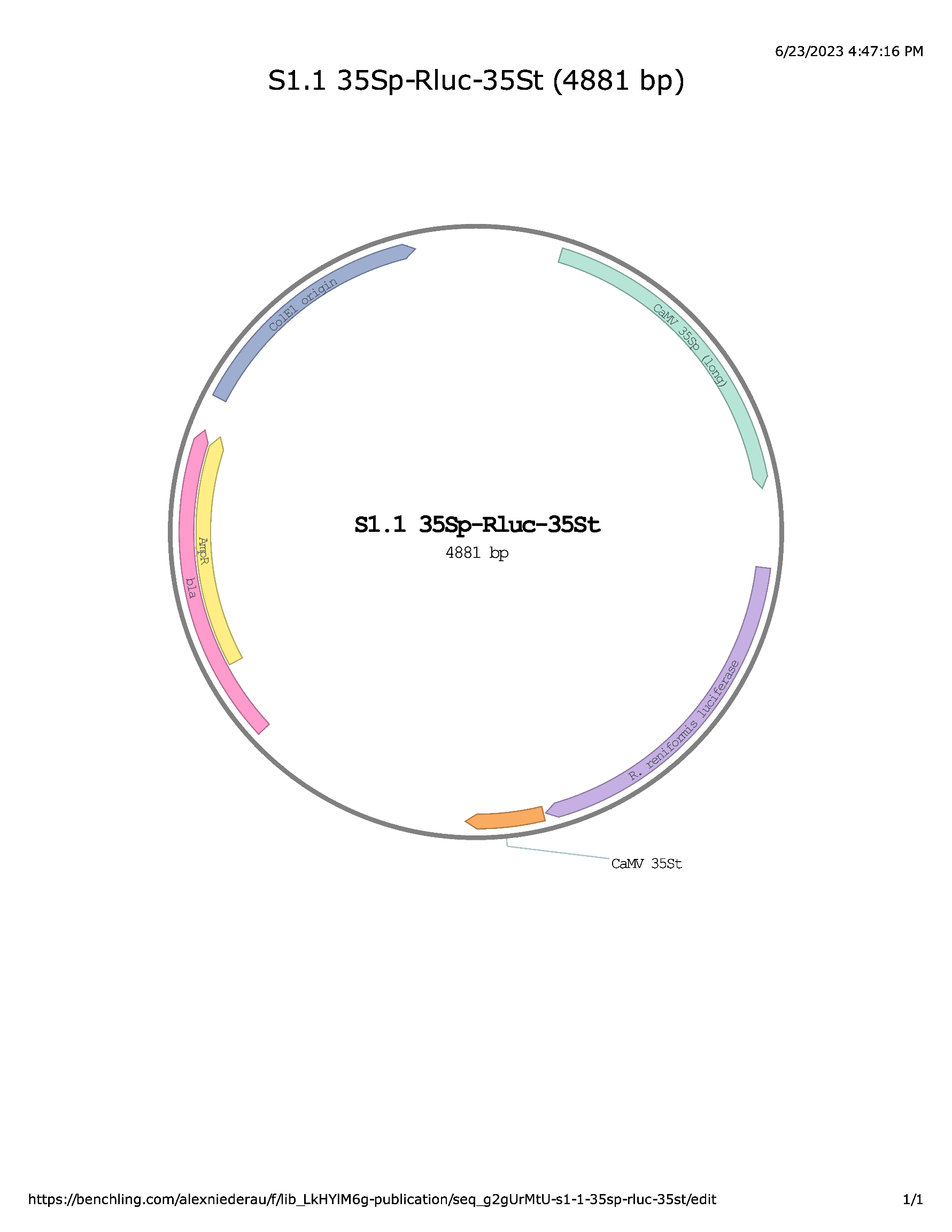


**Fig. S1.1 Vector map of 35Sp-Rluc-35St.**

*Renilla reniformis* luciferase-encoding vector used as a transformation control throughout experiments.


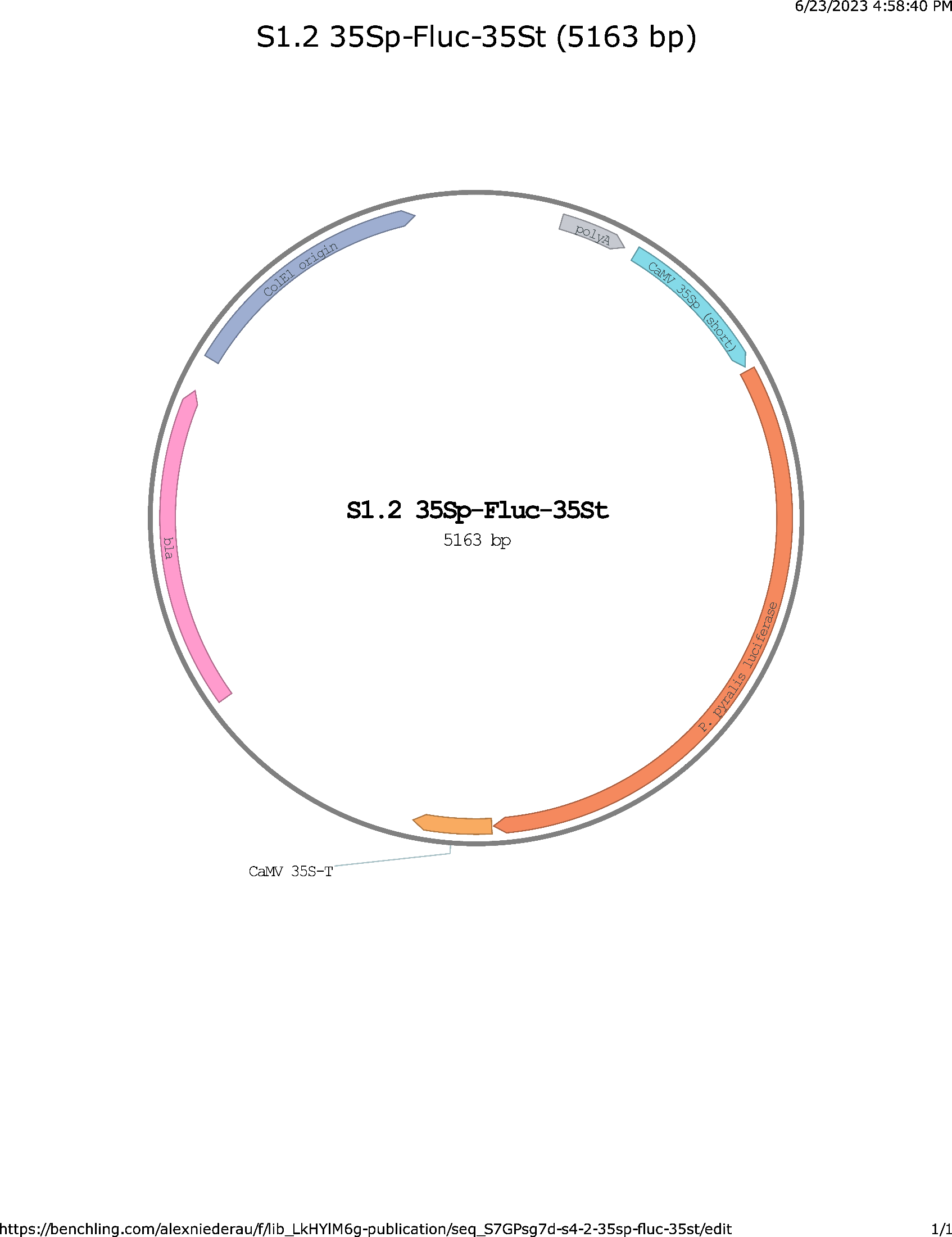


**Fig. S1.2 Vector map of 35Sp-Fluc-35St.**

*Photinus pyralis* firefly luciferase-encoding vector used as a reference throughout experiments and as a template for cloning of terminator testing constructs.


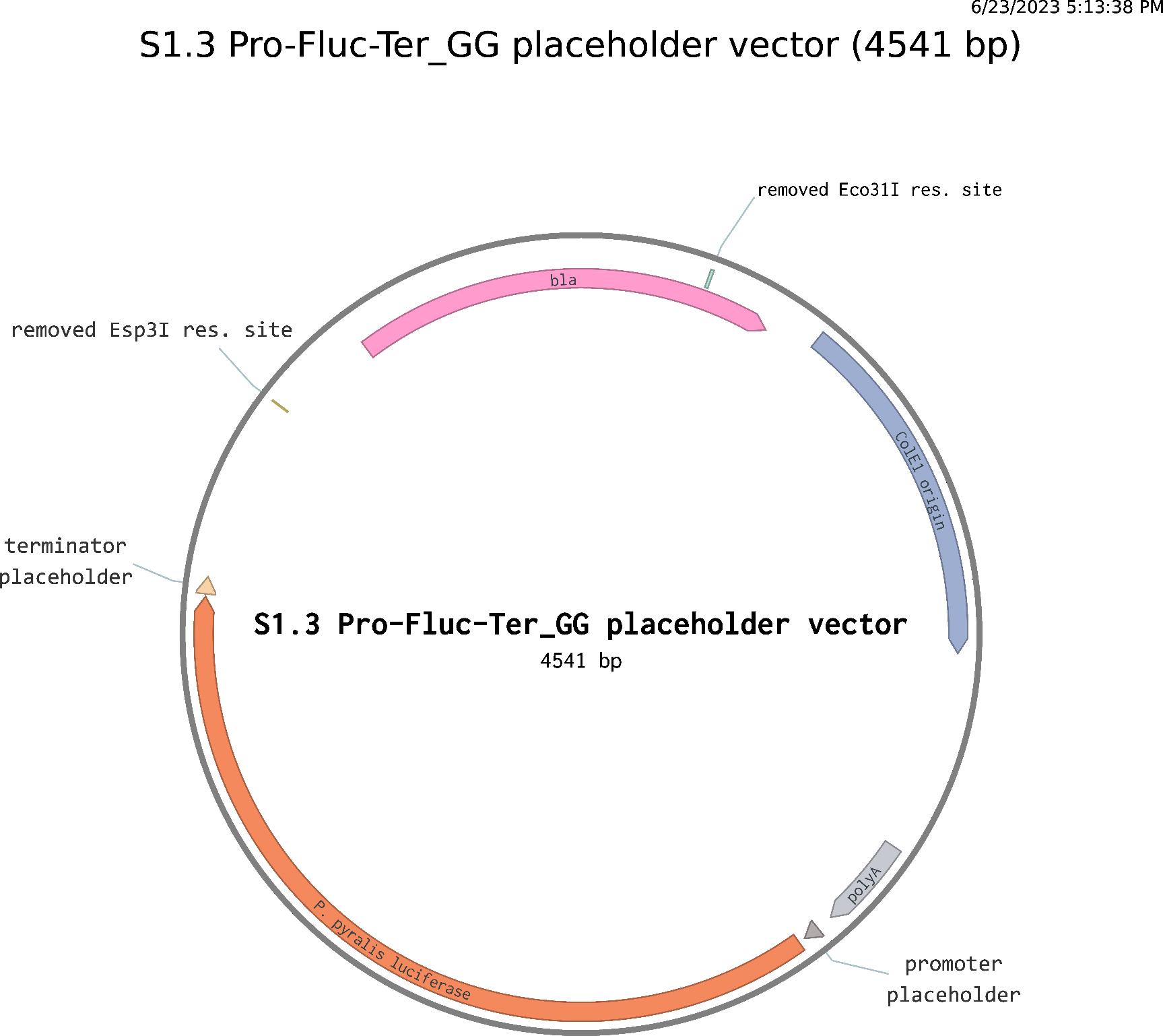


**Fig. S1.3 Vector map of Pro-Fluc-Ter_GG placeholder vector.**

*Photinus pyralis* firefly luciferase-encoding vector used as a template for cloning of terminator testing constructs via the Golden Gate-like cloning approach.

**Supplementary table S2 - List of primers**

Cloning of single terminator sequences via restriction-ligation reactions

| Primer Name | Sequence (5‘ -> 3‘) |
| --- | --- |
| Cab1/Fw | ACTAGATCTATTTTTGTGCTTATGCAGCTATGAC |
| Cab1/Rv | ACTCTGCAGGATCTTTCGCAATTTGTGCGATAG |
| dehyd/Fw | ACTAGATCTGCGCCGATTGTGTATAACTCAC |
| dehyd/Rv | ACTCTGCAGCACCGAAATCGATCATCGTTCAC |
| petE/Fw | ACTAGATCTAGGGTTTGATTTGTTGTCTTCTCTTC |
| petE/Rv | ACTCTGCAGAGAAGACGGTAACGAGCCTAATG |
| Cab2/Fw | ACTAGATCTAGTGACCGTTCATTGTAAATACCTC |
| Cab2/Rv | ACTCTGCAGGGATTGTGATGTGAGACAGTGAC |
| Catalase/Fw | ACTAGATCTACCATCTGTTTATTTTAAATCATATTTTAAGTATGTCTAC |
| Catalase/Rv | ACTCTGCAGCAACCGCAAATGATTTCAAATTCTC |
| PSII/Fw | ACTAGATCTACTTGTATACTCTCCGTCTCGC |
| PSII/Rv | ACTCTGCAGTATCATGAGTTTGATTGGTATGTTTACC |
| UK/Fw | ACTAGATCTGTATGTGCATACGCATTAGCTTTTG |
| UK/Rv | ACTCTGCAGTAGTGACATGGCTCTC |
| rbcS-T/Fw | AGAAGGGCGGAAAGATCGCCGTGTAAGATCTATACTTCAGTCGAGGCCATG |
| rbcS-T/Rv | TAAAACGACGGCCAGTGCCAAGCTTCTGCAGCACATGTTTGATAGGAGAATAGGTTAAAG |
| EF1A-T/Fw | ACTAGATCTATAGTGCGGGATTTGGTTTTG |
| EF1A-T/Rv | ACTCTGCAGGGGACATTTCTTCAGCTATTAGTAC |
| psaE-T/Fw | ACTAGATCTGGCAGTTTAGCCTCTCTTG |
| psaE-T/Rv | ACTCTGCAGTACATATATGTAGACACTGCGC |
| RP-L18A-T/Fw | ACTAGATCTAAAAACAGTCGAGAAGGATCC |
| RP-L18A-T/Rv | ACTCTGCAGAGGAGACTTGTAAAATTTGTAGCC |
| RP-S6e-T/Fw | ACTAGATCTATTCTCATGGATTTTTCTGAAGTAGTTC |
| RP-S6e-T/Rv | ACTCTGCAGGAGCATAATATCAAAGACAGATGTGC |
| RP-S18e-T/Fw | ACTAGATCTAGTTTTTAATCTTTTGTCTGAAGGG |
| RP-S18e-T/Rv | ACTCTGCAGACGTTTGATATCTTACGTGTGG |
| int.lessEXT/Fw | ACTAGATCTAAAGCAGAATGCTGAGCTAAAAG |
| int.lessEXT /Rv | ACTCTGCAGGTCATAACTGTAGAAATGATTCCATTAC |

Cloning of promoter sequences via one pot Golden Gate-like reactions

| Primer Name | Sequence (5‘ -> 3‘) |
| --- | --- |
| PpAct5p_GG_Fw | CAGCGTCTCTCTAGATCCTTGTGACTTTTGTGCATTATTGAG |
| PpAct5p_GG_Rv | GGACGTCTCCTCGAGGCTGCACAACAACCACCAATATTTAATTTC |
| CaMV35Sp_GG_Fw | CAGCGTCTCTCTAGATCGACAACATGGTGGAGCAC |
| CaMV35Sp_GG_Rv | GGACGTCTCCTCGAGGGTCCTCTCCAAATGAAATGAACTTC |
| NOSp_GG_Fw | CAGCGTCTCTCTAGAGAACCGCAACGTTGAAGGAG |
| NOSp_GG_Rv | GGACGTCTCCTCGAGTAATTGGATACCGAGGGGAATTTATGG |

Cloning of single terminator sequences via one pot Golden Gate-like reactions

| Primer Name | Sequence (5‘ -> 3‘) |
| --- | --- |
| PpCab1t_GG_Fw | CACGGTCTCAGATCTATTTTTGTGCTTATGCAGCTATG |
| PpCab1t_GG_Rv | GTCGGTCTCTGCAGGATCTTTCGCAATTTG |
| Ppdehydt_GG_Fw | CACGGTCTCAGATCTGCGCCGATTGTG |
| Ppdehydt_GG_Rv | GTCGGTCTCTGCAGCACCGAAATCG |
| PppetEt_GG_Fw | CACGGTCTCAGATCTAGGGTTTGATTTGTTGTCTTC |
| PppetEt_GG_Rv | GTCGGTCTCTGCAGAGAAGACGGTAACG |
| PpCab2t_GG_Fw | CACGGTCTCAGATCTAGTGACCGTTCATTGTAAATACC |
| PpCab2t_GG_Rv | GTCGGTCTCTGCAGGGATTGTGATGTGAG |
| PprbcSt_GG_Fw | CACGGTCTCAGATCTATACTTCAGTCGAGGCC |
| PprbcSt_GG_Rv | GTCGGTCTCTGCAGCACATGTTTGATAGG |
| PpRP-S6e_GG_Fw | CACGGTCTCAGATCTATTCTCATGGATTTTTCTGAAGTAGTTCTTC |
| PpRP-S6e_GG_Rv | GTCGGTCTCTGCAGGAGCATAATATCAAAGACAGATGTGCTTAC |
| PpnoInt-UK2_GG_Fw | CACGGTCTCAGATCTAACTCGTTTCTTACCTCACACTGGG |
| PpnoInt-UK2_GG_Rv | GTCGGTCTCTGCAGTTTTACTTTAGGAGACTGTCTTTACAGGTTTTAAATG |
| CaMV35St_GG_Fw | CACGGTCTCAGATCTGTCCGCAAAAATCACC |
| CaMV35St_GG_Rv | GTCGGTCTCTGCAGGTCACTGGATTTTG |
| NbEUt_GG_Fw | CACGGTCTCAGATCTAAAGCAGAATGCTGAGC |
| NbEUt_GG_Rv | GTCGGTCTCTGCAGGTCATAACTGTAGAAATG |
| AtHSP90t_GG_Fw | CACGGTCTCAGATCTATATGAAGATGAAGATGAAATATTTGGTGTGTC |
| AtHSP90t_GG_Rv | GTCGGTCTCTGCAGGCGATTCCCGATACG |
| NOSt_GG_Fw | CAAAAATGAACAATAAACTAGTGATCGTTCAAACATTTGGCAATAAAGTTTC |
| NOSt_GG_Rv | GTCGGTCTCTGCAGGATCTAGTAACATAGATGACACCGCG |

Cloning of double terminator constructs

| Primer Name | Sequence (5‘ -> 3‘) |
| --- | --- |
| 35ST_BglII_F | GTAGATCTGTCCGCAAAAATCACCAGTC |
| 35ST_BglII_R | GTAGATCTGTCACTGGATTTTGGTTTTAGGA |
| NosT_PstI_F | TTCTGCAGCCCCGATCGTTCAAACATT |
| NosT_PstI_R | TGCTGCAGACCCGATCTAGTAACATAGATG |
| NosT_BglII_F | GTAGATCTCCCCGATCGTTCAAACATT |
| NosT_BglII_R | CCAGATCTACCCGATCTAGTAACATAGATG |
| 2xNosT_F | TATGTTACTAGATCGGGTCCCCGATCGTTCAAACATT |
| 2xNosT_R | TGTTTGAACGATCGGGGACCCGATCTAGTAACATAGATG |
| petE_dT/Fw | ACTCTGCAGAGGGTTTGATTTGTTGTCTTCTCTTC |
| Cab2_dT/Fw | ACTCTGCAGAGTGACCGTTCATTGTAAATACCTC |
| RP-S6e_GG_dT1_Rv | GTCGGTCTCTGCCGGAGCATAATATCAAAGACAGATGTGCTTAC |
| RP-S6e_GG_dT2_Fw | CACGGTCTCTCGGCAGATTCTCATGGATTTTTCTGAAGTAGTTCTTCTGTTCTAGTAATG |
| AtHSP90t_GG_dT1_Rv | GTCGGTCTCTGCCGGCGATTCCCGATACGTAGTCTC |
| AtHSP90t_GG_dT2_Fw | CACGGTCTCTCGGCAGATATGAAGATGAAGATGAAATATTTGGTGTGTCAAATAAAAAG |

Cloning of Pro-Fluc-Ter_GGplaceholder vector

| Primer Name | Sequence (5‘ -> 3‘) |
| --- | --- |
| Ampl.1_5OH-ter.ph_Fw | GACCTCCAGAATCGTAGTGCCTGAGGTCTCACTGCAGAAGCTTGGCACTGG |
| Ampl.1_3OH-ampl.2_Rv | CGAGGCCCTTTCGTCTGACGGTCACAGCTTGTCTGTAAG |
| Ampl.2_5OH-ampl.1_Fw | ACAAGCTGTGACCGTCAGACGAAAGGGCCTCGTGATAC |
| Ampl.2_3OH-ampl.3_Rv | GATACCGCGAGAGCCACGC |
| Ampl.3_5OH-ampl.2_Fw | GAGCGTGGCTCTCGCG |
| Ampl.3_3OH-pro.ph_Rv | CGTGCAGATACGTGACAGCTAGCGTCTCTCTAGAGGATCCCCGGGTAC |
| Ampl.4_5OH-pro.ph_Fw | CGCTAGCTGTCACGTATCTGCACGTCTCCTCGAGCTCAACCATGGAAG |
| Ampl.4_3OH-ter.ph_Rv | CCTCAGGCACTACGATTCTGGAGGTCTCAGATCTTACACGGCGATCTTTCC |

**Supplementary table S3 - List of vectors used in this study**

Vectors used from Horstman et al. (2004)

| No° | Vector name |
| --- | --- |
| 1 | 35Sp-Fluc-35St |
| 2 | 35Sp-Rluc-35St |

Vectors cloned as part of this study

| No° | Vector name |
| --- | --- |
| 3 | 35Sp-Fluc-Cab1t |
| 4 | 35Sp-Fluc-dehydt |
| 5 | 35Sp-Fluc-petEt |
| 6 | 35Sp-Fluc-Cab2t |
| 7 | 35Sp-Fluc-Catalaset |
| 8 | 35Sp-Fluc-PSIIt |
| 9 | 35Sp-Fluc-UKt |
| 10 | 35Sp-Fluc-rbcSt |
| 11 | 35Sp-Fluc-EF1At |
| 12 | 35Sp-Fluc-psaEt |
| 13 | 35Sp-Fluc-RPL18at |
| 14 | 35Sp-Fluc-RPS6et |
| 15 | 35Sp-Fluc-RPS18et |
| 16 | 35Sp-Fluc-noIUK2t |
| 17 | 35Sp-Fluc-EUt |
| 18 | 35Sp-Fluc-HSPt |
| 19 | 35Sp-Fluc-NOSt |
| 20 | NOSp-Fluc-Cab1t |
| 21 | NOSp-Fluc-dehydt |
| 22 | NOSp-Fluc-petEt |
| 23 | NOSp-Fluc-Cab2t |
| 24 | NOSp-Fluc-rbcSt |
| 25 | NOSp-Fluc-RPS6et |
| 26 | NOSp-Fluc-noIUK2t |
| 27 | NOSp-Fluc-35St |
| 28 | NOSp-Fluc-EUt |
| 29 | NOSp-Fluc-HSPt |
| 30 | NOSp-Fluc-NOSt |
| 31 | Act5p-Fluc-Cab1t |
| 32 | Act5p-Fluc-dehydt |
| 33 | Act5p-Fluc-petEt |
| 34 | Act5p-Fluc-Cab2t |
| 35 | Act5p-Fluc-rbcSt |
| 36 | Act5p-Fluc-RPS6et |
| 37 | Act5p-Fluc-noIUK2t |
| 38 | Act5p-Fluc-35St |
| 39 | Act5p-Fluc-EUt |
| 40 | Act5p-Fluc-HSPt |
| 41 | Act5p-Fluc-NOSt |
| 42 | 35Sp-Fluc-35St35St |
| 43 | 35Sp-Fluc-NOStNOSt |
| 44 | 35Sp-Fluc-35StNOSt |
| 45 | 35Sp-Fluc-NOSt35St |
| 46 | 35Sp-Fluc-Cab2tCab2t |
| 47 | 35Sp-Fluc-petEtpetEt |
| 48 | 35Sp-Fluc-Cab2tpetEt |
| 49 | 35Sp-Fluc-petEtCab2t |
| 50 | 35Sp-Fluc-S6etS6et |
| 51 | 35Sp-Fluc-HSPtHSPt |
| 52 | 35Sp-Fluc-S6etHSPt |
| 53 | 35Sp-Fluc-HSPtS6et |
| 54 | Pro-Fluc-Ter_GGplaceholder |

| **Supplementary file S7 - Principal Component Analysis of reporter values and terminator attributes**. | | | | | | | | |
| --- | --- | --- | --- | --- | --- | --- | --- | --- |
| Combination | reporter values | poly(A) sites | PAS | expression levels (FPKM) | length (bp) | Promoter | Cluster | uncertainty |
| 35Sp35St | 1 | 2 | 1 | N/A | 211 | 35Sp | 1 | 3.40E-13 |
| 35SpNOSt | 0.806579 | 3 | 1 | N/A | 253 | 35Sp | 1 | 1.46E-09 |
| 35SppetEt | 0.555284 | 2 | 0 | 1178.96 | 334 | 35Sp | 1 | 2.44E-10 |
| 35SpRPS18et | 0.437111 | 2 | 1 | 213.41 | 326 | 35Sp | 1 | 5.35E-10 |
| 35SprbcSt | 0.314961 | 2 | 0 | 749.16 | 339 | 35Sp | 1 | 1.25E-11 |
| 35SpEF1At | 0.201864 | 3 | 0 | 535.83 | 433 | 35Sp | 1 | 0.000247567 |
| 35SpCatalaset | 0.146145 | 1 | 0 | 879.7 | 317 | 35Sp | 1 | 2.22E-16 |
| 35SpPSIIt | 0.142066 | 2 | 0 | 866.63 | 306 | 35Sp | 1 | 9.10E-15 |
| 35SpUKt | 0.139687 | 3 | 0 | 834.25 | 374 | 35Sp | 1 | 2.68E-08 |
| Act5pNOSt | 1.092518 | 3 | 1 | N/A | 253 | Act5p | 1 | 1.15E-07 |
| Act5p35St | 1 | 2 | 1 | N/A | 211 | Act5p | 1 | 3.40E-13 |
| Act5ppetEt | 0.699023 | 2 | 0 | 1178.96 | 334 | Act5p | 1 | 2.18E-09 |
| Act5prbcSt | 0.653876 | 2 | 0 | 749.16 | 339 | Act5p | 1 | 2.19E-09 |
| NOSp35St | 1 | 2 | 1 | N/A | 211 | NosP | 1 | 3.40E-13 |
| NOSppetEt | 0.874447 | 2 | 0 | 1178.96 | 334 | NosP | 1 | 3.17E-08 |
| NOSpNOSt | 0.823123 | 3 | 1 | N/A | 253 | NosP | 1 | 1.89E-09 |
| NOSprbcSt | 0.792393 | 2 | 0 | 749.16 | 339 | NosP | 1 | 1.82E-08 |
| 35SpHSPt | 1.101886 | 8 | 3 | N/A | 464 | 35Sp | 2 | 0.000312678 |
| 35SpCab2t | 0.720161 | 9 | 0 | 884.36 | 611 | 35Sp | 2 | 4.06E-06 |
| 35SpEUt | 0.700964 | 9 | 2 | N/A | 480 | 35Sp | 2 | 0.00332314 |
| Act5pHSPt | 1.609497 | 8 | 3 | N/A | 464 | Act5p | 2 | 7.43E-07 |
| Act5pEUt | 1.274643 | 9 | 2 | N/A | 480 | Act5p | 2 | 3.61E-06 |
| Act5pCab2t | 1.238835 | 9 | 0 | 884.36 | 611 | Act5p | 2 | 8.46E-09 |
| NOSpCab2t | 1.073199 | 9 | 0 | 884.36 | 611 | NosP | 2 | 6.08E-08 |
| NOSpHSPt | 1.018311 | 8 | 3 | N/A | 464 | NosP | 2 | 0.000845051 |
| NOSpEUt | 0.88378 | 9 | 2 | N/A | 480 | NosP | 2 | 0.000378276 |
| 35SpRPS6et | 0.814261 | 6 | 1 | 213.83 | 476 | 35Sp | 3 | 0.00027916 |
| 35SpnoIUK2t | 0.786362 | 7 | 3 | 82.39 | 393 | 35Sp | 3 | 0.001580687 |
| 35SpCab1t | 0.602424 | 5 | 1 | 5428.09 | 456 | 35Sp | 3 | 2.19E-07 |
| 35Spdehydt | 0.390909 | 5 | 0 | 2382.07 | 503 | 35Sp | 3 | 2.55E-07 |
| 35SpRPL18at | 0.336527 | 5 | 1 | 288.67 | 447 | 35Sp | 3 | 3.11E-05 |
| 35SppsaEt | 0.268661 | 6 | 0 | 529.37 | 486 | 35Sp | 3 | 1.17E-07 |
| Act5pRPS6et | 1.398167 | 6 | 1 | 213.83 | 476 | Act5p | 3 | 0.225578858 |
| Act5pdehydt | 0.78077 | 5 | 0 | 2382.07 | 503 | Act5p | 3 | 2.32E-06 |
| Act5pCab1t | 0.763587 | 5 | 1 | 5428.09 | 456 | Act5p | 3 | 4.51E-07 |
| Act5pnoIUK2t | 0.475473 | 7 | 3 | 82.39 | 393 | Act5p | 3 | 3.91E-05 |
| NOSpdehydt | 1.023659 | 5 | 0 | 2382.07 | 503 | NosP | 3 | 4.17E-05 |
| NOSpRPS6et | 0.828497 | 6 | 1 | 213.83 | 476 | NosP | 3 | 0.000330689 |
| NOSpCab1t | 0.806717 | 5 | 1 | 5428.09 | 456 | NosP | 3 | 7.39E-07 |
| NOSpnoIUK2t | 0.739526 | 7 | 3 | 82.39 | 393 | NosP | 3 | 0.000905805 |

Column 1 contains the different promoter-terminator combinations included in the PCA. Column 2, 3, 4, and 5 contain the terminator attributes included in the Principal Component Analysis. Column 8 gives the assigned cluster, also indicated by colour. Column 9 contains the uncertainty value for the cluster assignment of each individual promoter-terminator combination.

| **Supplementary file S8: Scores of the PCA of reporter values and terminator attributes**. combination | Comp.1 | Comp.2 |
| --- | --- | --- |
| 35Sp35St | -1.27271 | 1.741122 |
| 35SpHSPt | 2.267145 | 1.010754 |
| 35SpRPS6et | 0.674693 | -0.409 |
| 35SpNOSt | -1.07776 | 1.167619 |
| 35SpnoIUK2t | 1.31021 | 1.122578 |
| 35SpCab2t | 1.50224 | -2.13901 |
| 35SpEUt | 1.686728 | -0.22925 |
| 35SpCab1t | 0.077048 | -0.47967 |
| 35SppetEt | -1.6678 | -0.13731 |
| 35SpRPS18et | -1.44992 | 0.328473 |
| 35Spdehydt | -0.37295 | -1.59894 |
| 35SpRPL18at | -0.29322 | -0.7586 |
| 35SprbcSt | -1.94302 | -0.46997 |
| 35SppsaEt | -0.35678 | -1.72115 |
| 35SpEF1At | -1.41321 | -1.26146 |
| 35SpCatalaset | -2.4964 | -0.47432 |
| 35SpPSIIt | -2.30663 | -0.48483 |
| 35SpUKt | -1.75726 | -0.97773 |
| Act5pHSPt | 2.896249 | 1.648622 |
| Act5pRPS6et | 1.39835 | 0.324743 |
| Act5pEUt | 2.397711 | 0.491641 |
| Act5pCab2t | 2.145052 | -1.48724 |
| Act5pNOSt | -0.72339 | 1.526931 |
| Act5p35St | -1.27271 | 1.741122 |
| Act5pdehydt | 0.110222 | -1.10904 |
| Act5pCab1t | 0.276783 | -0.27716 |
| Act5ppetEt | -1.48966 | 0.043313 |
| Act5prbcSt | -1.52299 | -0.04408 |
| Act5pnoIUK2t | 0.924912 | 0.731912 |
| NOSpCab2t | 1.939774 | -1.69538 |
| NOSpdehydt | 0.411244 | -0.80382 |
| NOSpHSPt | 2.163568 | 0.905734 |
| NOSp35St | -1.27271 | 1.741122 |
| NOSpEUt | 1.9133 | 0.00048 |
| NOSppetEt | -1.27225 | 0.263751 |
| NOSpRPS6et | 0.692336 | -0.39111 |
| NOSpNOSt | -1.05726 | 1.188408 |
| NOSpCab1t | 0.330237 | -0.22296 |
| NOSprbcSt | -1.35132 | 0.129976 |
| NOSpnoIUK2t | 1.252164 | 1.063724 |

Column 1 contains the different promoter-terminator combinations included in the PCA. Column 2 & 3 contain the scores for principal component no. 1 & no. 2, respectively.
